# Host–Parasite Coevolution in Primates

**DOI:** 10.3390/life13030823

**Published:** 2023-03-17

**Authors:** Dietmar Zinner, Filipa M. D. Paciência, Christian Roos

**Affiliations:** 1 Cognitive Ethology Laboratory, German Primate Center, Leibniz Institute for Primate Research, 37077 Göttingen, Germany; 2 Department of Primate Cognition, Georg-August-University of Göttingen, 37077 Göttingen, Germany; 3 Leibniz Science Campus Primate Cognition, 37077 Göttingen, Germany; 4 AAP Rescue Centre for Exotic Animals, 1358 AC Almere, The Netherlands; 5 Gene Bank of Primates and Primate Genetics Laboratory, German Primate Center, Leibniz Institute for Primate Research, 37077 Göttingen, Germany

**Keywords:** infectious disease, virulence, co-speciation, phylogeny

## Abstract

Organisms adapt to their environment through evolutionary processes. Environments consist of abiotic factors, but also of other organisms. In many cases, two or more species interact over generations and adapt in a reciprocal way to evolutionary changes in the respective other species. Such coevolutionary processes are found in mutualistic and antagonistic systems, such as predator–prey and host–parasite (including pathogens) relationships. Coevolution often results in an “arms race” between pathogens and hosts and can significantly affect the virulence of pathogens and thus the severity of infectious diseases, a process that we are currently witnessing with SARS-CoV-2. Furthermore, it can lead to co-speciation, resulting in congruent phylogenies of, e.g., the host and parasite. Monkeys and other primates are no exception. They are hosts to a large number of pathogens that have shaped not only the primate immune system but also various ecological and behavioral adaptions. These pathogens can cause severe diseases and most likely also infect multiple primate species, including humans. Here, we briefly review general aspects of the coevolutionary process in its strict sense and highlight the value of cophylogenetic analyses as an indicator for coevolution.

## 1. Introduction

The mammalian order of primates consists of two suborders: Strepsirrhini (lemurs, galagos, and lorisids) and Haplorhini (tarsiers, monkeys, and apes—which include humans). Like any other organism, primates constitute ecosystems for a large number of organisms, living on or in primate hosts, e.g., microbes of the skin or the gut. Depending on the effects on the host’s Darwinian or evolutionary fitness (see Table 1), the relationships between these organisms and the host can be classified as mutualism (beneficial for both sides), commensalism (beneficial for the colonizing organism, but neutral for the host), or parasitism (beneficial for the colonizing organism, negative for the host). Parasites feed and grow in or on the host or use their cellular processes to survive and multiply. Viruses, bacteria, protozoa, helminths, and arthropods can have significant negative effects on the fitness and health of the host [1]. If these negative effects can cause disease in the host, a parasite can be regarded as a pathogen. However, not all parasites cause disease, although they strongly affect the host’s fitness, e.g., brood parasites such as the cuckoo (*Cuculus canorus*). In the following review, we will mainly focus on pathogens and will use the terms parasite and pathogen partly interchangeably.

Pathogens are part of the natural habitats of wild primates and since at least anthropoid primates (monkeys and apes) share a similar physiology, many of these pathogens have the potential to cross species boundaries relatively easily, which also includes transmission between humans and nonhuman primates, and vice versa [2,3]. The closer the phylogenetic relationships among possible host species are, the more likely a transmission, given that there is contact. Pathogens, which exclusively infect a particular primate species, may even evolve into forms that exclusively infect humans [4]. However, in primates, as in other mammals, our understanding of the coevolutionary processes between hosts and parasites is still limited [5].

Primates, like other organisms, have evolved in heterogeneous environments, which pose a multitude of challenges to their survival and reproduction. Among individuals, there is genetic, and as a result, phenotypic variation, resulting in some individuals leaving more offspring than others. They achieve higher Darwinian fitness and the respective alleles spread in subsequent populations. Thus, these environmental conditions constitute selective pressures, and the progeny of the individuals can be regarded as better adapted [6].

An ecosystem is composed of abiotic factors (e.g., climate and soil chemistry) and biotic factors (living organisms). Organisms do not live in isolation and interactions with other taxa are ubiquitous and an important feature of the environment. Animals feed on plants or other animal species, compete for resources, are threatened by predators, and are also infested by parasites. They provide the habitat for billions of microorganisms living on and inside their bodies, as commensals, mutualists, or parasites [7,8]. In other words, organisms evolve in response to the interaction with other species. These interactions can have different fitness effects on the participating organisms and are classified accordingly [9]. In the case of mutualism, individuals of both interacting species benefit from their interaction, in case of competition, individuals of both species lose fitness, and in cases of predation and parasitism, individuals of one species gain fitness at the fitness expense of individuals of the other species. Commensalism occurs if one species increases its fitness without any consequences for the second species, and in many cases, the interactions between the species are just neutral, without any effect on the fitness of either species.

In many cases, an evolutionary change in one species triggers an evolutionary response in a second species, including parasites and pathogens, which again leads to a trait change in the first species or host. This means that changes in gene frequencies as a result of selection acting on one population (species) creates selection pressure for changes in gene frequencies in the other population (species), which again creates selection in the first population (species), and so on [10]. Such reciprocal evolutionary changes in two or more species have been defined as coevolution in a strict sense [11].

Coevolution among species plays a major role in the evolution and maintenance of a wide array of biological phenomena, including local adaptation, speciation, and resistance against parasites. Although it has proven difficult to rigorously demonstrate coevolution in practice, it can have a major influence on how genetic variation in biologically and biomedically important traits (e.g., immune system) is interpreted, and thus can contribute to our understanding of health and disease [10]. With our review, we aim to provide a brief overview of the various aspects of coevolution and will discuss some examples of possible coevolution in relation to infectious diseases in nonhuman primates.

**Table 1:** Glossary ^1^.

Adaptation	Genetic change that increases fitness (operationally reproductive success) in response to natural selection
Co-adaptation	Reciprocal adaptation in two or more ecologically interacting species, such as a host and its parasite (pathogen) or a flowering plant and its pollinator
Coevolution	Joint evolution of two or more ecologically interacting species. Often used in the strict sense as an equivalent to co-adaptation
Co-diversification	Joint diversification of interacting lineages: (1) early step in the process of co-speciation, and (2) more suitable term than co-speciation in cases where species delimitation is difficult (e.g., bacterial strains, viruses)
Co-speciation	Joint speciation of both the host and parasite. Either the host or parasite may speciate slightly after or before the other
Evolutionary Arms Race	A struggle between competing sets of coevolving genes, traits, or species, that leads to adaptations and counter-adaptations against each other
Fahrenholz’s Rule	Parasite phylogeny mirrors host phylogeny
Fitness	Individual reproductive success. Equal to the average contribution of an individual to the gene pool of the next generation(s)
Virulence	Parasite (pathogen)-induced reduction in host fitness, and the ability of a parasite to cause disease in and/or death of its hosts (morbidity and mortality)

^1^ [6,9,12,13,14].

## 2. Coevolution

Ehrlich and Raven [15] were the first to coin the term coevolution in a study on the effects of secondary chemical compounds in plants acting as defense substances on herbivore butterflies (butterflies whose larvae feed on these plants). However, Charles Darwin [16] already mentioned evolutionary interactions between flowering plants and insects as pollinators. He developed this idea further and, considering the 30 cm-long nectar spur of the Malagasy star orchid (*Angraecum sesquipedale*), predicted that there must be a pollinator adapted to this long spur [17]. Forty years later, Morgan’s sphinx moth (*Xanthopan morganii*) was discovered and identified as the pollinator, which indeed has a proboscis that is also about 30 cm-long [18]. Another intriguing mutualistic coevolution can be found between figs and their pollinators, the fig wasps of the family Agaonidae [19,20]. Each of the more than 750 species of figs has its own pollinating wasp species, and this specificity is maintained by responses of the wasps to fig volatile cues [21,22].

Coevolution in the strict sense refers to pairwise interactions among species, e.g., reciprocal trait changes in two species, A and B, can be detected. An evolutionary change in species A is followed by a change in species B, which in turn triggers a second change in species A, and so on (evolutionary “arms race”). In many cases, however, the situation is more complicated since one species interacts with multiple species and it may come to diffuse coevolution, i.e., multiple interacting species have influenced one another in some reciprocal manner [6]. For instance, many pollinator species interact with several plant species. Honeybees (*Apis mellifera*) and many related Hymenoptera are not specialized on only one plant species but visit and pollinate a number of species [23]. In addition, when it comes to seed dispersal in plants, multi-species interactions can be observed. Within an ecological community, a whole guild of frugivorous animals (e.g., primates, birds, bats) are potential seed dispersers. Plants, thus, may co-adapt in fruit and seed characteristics to the whole guild or just to a few specialized frugivores. Frugivores, on the other hand, may co-adapt to certain fruit and seed characteristics [24]. The evolutionary interaction of multiple species that have influenced one another in some reciprocal evolutionary way is called diffuse coevolution [25] and may also be the usual situation when considering primates and their pathogens.

Concerning pollination and in contrast to seed dispersal, there is a certain selection pressure on the plant part to make sure that the pollinator sticks to the same plant species when carrying pollen from one flower to the next. The pollen delivery system must be more precise than the seed dispersal system. Therefore, one finds tighter co-adaptations among plants and pollinators than among plants and seed dispersers [26].

**Figure 1:**
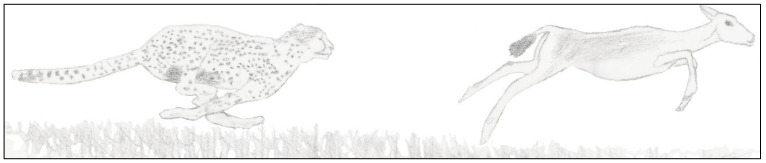
Predator–prey interaction. Cheetah (*Acinonyx jubatus*) chasing an impala (*Aepyceros melampus*). Drawing by David Zinner.

Interactions such as the ones between plants, pollinators, and seed dispersers or the ability of folivorous primates such as colobine monkeys to digest food items with a high fiber content with the help of their gut microbiota [27], are examples of mutualistic relationships. In contrast, an example of antagonistic interactions is the evolution of a predator–prey relationship. A prey species evolves to run faster than the predator, which exerts selection pressure on the predator to run even faster (Figure 1). In turn, the prey species evolves a response to the faster predator, and so on. Such processes of adaptation and counter-adaptation may continue in a positive feedback loop until costs outweigh benefits or when an energetic or physiological threshold is reached [28]. Similar coevolution can occur between parasites and hosts. Such antagonistic processes may result in unstable runaway escalations or perpetual “arms races”, where one side evolves mechanisms to avoid predation or parasitism, which is then countered by more sophisticated armament or tactics to overcome the newly evolved defenses and tactics of prey or hosts [29]. An interesting case is the brood parasitism of the common cuckoo. Here, host species (small passerine birds) can be observed to mob, attack, and kill the cuckoo [30]. Adult common cuckoos resemble in appearance the Eurasian sparrowhawk (*Accipiter nisus*), which is a common predator of cuckoo hosts. It is hypothesized that cuckoos have evolved this mimicry to avoid such attacks. However, observations on a common host species, the red-backed shrike (*Lanius collurio*) suggest that this mimicry is no longer as effective as before [31].

Although there is continual evolutionary change in both species involved, the relationship between the two species will not change, i.e., normally, none of the species will evolutionarily outrun the other, i.e., drive it to extinction. The idea here is that species have to “run” (evolve) in order to stay in the same place (extant). Van Valen [32] dubbed it the Red Queen hypothesis.

**Figure 2:**
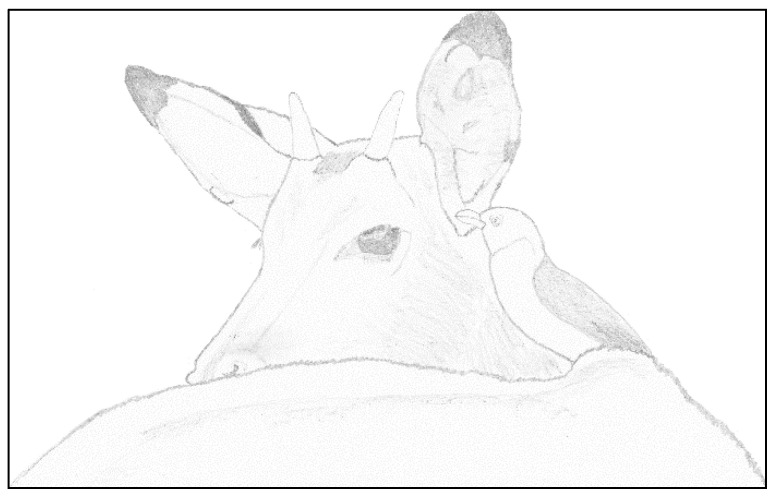
Oxpecker (*Buphagus* sp.) on a young impala (*Aepyceros melampus*). Drawing by David Zinner.

Biotic factors play important roles as selective pressures for all organisms in majority of cases and the term coevolution has been applied to various kinds of evolutionary interactions among organisms. It is even applied to non-biological phenomena, such as “coevolution” between supermassive black holes and star formation [33]. However, not every adaptation to another species can be regarded as coevolution. For instance, birds of the genus *Buphagus* (oxpeckers) are adapted to feed on ectoparasites of large ungulates of the African savannah (Figure 2). Thus, they provide some service to these ungulates by removing parasites, e.g., ticks. Although both species, the bird and the ungulate, obviously gain benefits from the birds’ foraging behavior, it remains questionable whether ungulates have responded evolutionarily to the interaction with oxpeckers. Here, the reciprocal evolutionary change might be missing, and the mutualistic relationship is most likely shaped by correlated evolution and not by coevolution [34].

Often, the term coevolution is also used in relation to evolutionary “arms races” within a single species (e.g., between sexes of the same species, sexual conflict [35,36]) or even within an individual (e.g., evolutionary changes within a population of parasites (viruses) within an individual host). Similar to these phenomena, the interdependent evolutionary reinforcement between brain size and the complexity of social systems in cetaceans and primates [37,38,39] does not, however, fulfil the strict definition of coevolution. In the case of sexual conflict and brain size evolution, the reciprocal adaptation does not occur between two populations or species, and in the case of viruses, the host represents more a kind of habitat for the parasite, and its immune system constitutes selection pressure for the parasite population. However, the individual host does not, and of course, cannot respond with an evolutionary change. Please note that viruses are regarded as obligate intracellular parasites [40].

## 3. Coevolution and Parasites

Intriguing examples of coevolution involve parasites and their hosts (e.g., [41]). However, host–parasite interactions do not always fulfil the criteria for coevolution, because sometimes selection acts only on one partner. For instance, a parasite may enter a small population of a host species, imposing selection and genetic change on the host population without being changed itself [42]. Additionally, if a new parasite invades a naïve host population, host mortality can be dramatic, e.g., the accidental introduction (in the 1960s) of a fly whose larvae suck blood from bird nestlings caused a mortality of 55% among nestling of Galapagos finches [43]. Similarly, the transmission of pathogens, such as pandemic human viruses, from humans into naïve great ape populations represents a major threat to these endangered species and is thus of great conservation concern [44,45].

The risk of infection by parasites represents a strong selective force and, as in predator–prey systems, evolutionary “arms races” can often be observed, where hosts evolve more and more elaborate behavioral, physiological, and immune defense mechanisms and parasites try to overcome this resistance [14]. The antagonistic coevolution between hosts and their parasites has been proposed as a fundamental driver of the genetic diversity found in populations [46,47,48], because parasites may cause frequency-dependent selection of rare genotypes (rare genotype advantage [41,42,49,50]). If a parasite invades a host population, it will most frequently encounter hosts with the most abundant genotypes in the population and will adapt to those genotypes. If they are adapted to one host genotype, they may be not able to infect hosts with other genotypes as well, making hosts with rare genotypes more resistant.

Parasites are believed to track specific host genotypes under natural conditions [51]. Since, by definition, pathogens negatively affect the host’s fitness, the former most frequent genotype will be reduced, whereas the frequency of hosts carrying the original rare genotype will increase and the parasite will adapt to the now most frequent genotype. Such frequency-dependent selection by parasites is hypothesized to be an important factor for the maintenance of polymorphisms of the immune system and indicates the potential for coevolution [52,53]. At least for humans, there is some evidence that exposure to certain forms of parasites during childhood is affecting the host’s immune system in a way that the risk for allergies and auto-immune diseases is reduced [54,55]. However, this does not contradict the general negative effect of pathogens.

Several parasites are known to exhibit multiple transmission modes, e.g., sexual and non-sexual contact. This is the case for instances of syphilis and yaws, genital and facial herpes, pubic and head lice, and genital and cutaneous warts. The duality in their transmission modes might be due to a single genotype having several transmission routes or that the parasite population contains several genetic strains, which are individually specialized for different routes of transmission [56].

In vertebrates, an adaptive immune system has evolved as a highly complex and multi-layered defense mechanism [57,58], the details of which are, however, beyond the scope of this review. It is even hypothesized that the risk of parasite infection drove local adaptation [47] and the prevalence of sexual reproduction over the more efficient asexual reproduction, as a measure to increase the genetic diversity within host populations, which would make it more difficult for parasites to attack a host [32,59,60].

In addition, in the host’s “meta-organism” (i.e., the body plus its microbiota [61]), the commensal or mutualistic species usually vastly outnumber the parasites, contributing to parasite defense and the regulation of the immune system, which can make coevolutionary processes rather complicated, because here, the large number of species involved makes coevolution more diffuse [62]. Similar diffuse coevolution can occur if a host population is infected by several parasite species. Studies with bacteria and their bacteriophages indicate that exposure to multiple parasites influences the rate and type of host–parasite coevolution [63]. An interesting question here is whether beneficial symbionts coevolve with their hosts and whether and how microbial parasites might have evolved into defensive symbionts [62].

## 4. Virulence

From an evolutionary perspective, hosts evolve in ways that minimize their fitness cost of being infected and parasites evolve to maximize their fitness by exploiting the host as an ecological resource. The effect of parasites on the host’s fitness can be more or less severe. Some parasites, e.g., lice, may take just little drops of blood from the host, but others may cause substantial mortality, e.g., viruses responsible for Ebola and yellow fever or bacteria causing anthrax in nonhuman primates [64]. Hence, by definition, the virulence of parasites differs. However, virulence not only differs among parasite species but also among host species. The same parasite can show different degrees of virulence in different host species. For instance, in various macaques (*Macaca* spp.) Macacine alpha-herpes virus 1 (Herpes B) causes symptoms similar to that of herpes simplex viruses (HSV) in humans. However, in humans, the same virus can lead to a severe central nervous system disease with a fatality rate of approximately 70% in untreated patients [65]. Other examples are immunodeficiency viruses. While the human immunodeficiency virus (HIV) causes AIDS in humans and the simian immunodeficiency virus of chimpanzees (SIVcpz) is pathogenic in free-ranging chimpanzees [66], other closely related SIVs, e.g., in African green monkeys (*Chlorocebus* spp.) or drills (*Mandrillus leucophaeus*), replicate efficiently in their natural hosts, without causing clinical symptoms [67,68]. Such observations suggest that species-specific host factors, such as interspecies differences in immune response, rather than intrinsic factors of the parasites, determine the virulence of the parasite [69].

Differential virulence is most likely a result of the coevolutionary interplay between the survival and reproductive strategies of the parasites and the various levels of the host’s defenses (e.g., behavioral, innate, and adaptive immune defenses). Parasites also have to migrate from one host to the next and high virulence might counteract high transmissibility, e.g., if a parasite that is sexually transmitted kills the host before the next sexual contact can occur. Therefore, one might expect sexually transmitted parasites to infect their hosts cryptically by yielding only minor cues of infection [70]. As a by-product, cryptic infections may also tend to be of relatively low virulence [71]. Thus, for HIV, high virulence as found in Ebola viruses would possibly not be an optimal strategy. Trade-offs between virulence and transmissibility influence the progression of parasite infections in individuals and populations [72]. Strong evidence for the trade-off model of virulence is also found in HIV. The question of why some infected host individuals develop AIDS rapidly whereas others remain healthy without treatment for many years is most likely related to the apparent conflict between the two levels of selection pressure on the virus [73,74]. On the other hand, if a sexually transmitted pathogen leads to symptoms perceivable (visible or olfactory) for potential sexual partners, sexual behavior and mate choice might be affected, as in the case of olive baboons (*Papio anubis*) infected with *Treponema pallidum* when females avoid mating with males showing visible signs of infection [75,76].

In some cases, when a new parasite invades a naïve population, virulence might be high in the beginning, whereas a long period of coexistence promotes adaptation of the host, and virulence will decline. However, this is not always the outcome of the “arms race” [77,78,79]. As most parasites reproduce within a host, some of their genotypes will have a selective advantage. They enable certain individuals to evade host defenses, reproduce better than others, and become more effective in utilizing the host’s resources, i.e., their negative effect on the host’s fitness will probably increase. They will become more virulent [14].

An interesting example where one can observe the evolution of a virus is SARS-CoV-2, a coronavirus that caused a pandemic. Transmission and virulence are largely decoupled for SARS-CoV-2 because transmission occurs during the course of infection long before severe or fatal consequences occur. Accordingly, it is also impossible to predict with certainty whether future virus variants will be more or less virulent than the currently (2022) prevalent omicron variants [80]. Considering the unpredictable nature of SARS-CoV-2 evolution, it is unclear whether future SARS-CoV-2 variants will derive primarily from omicron variants or from phylogenetically divergent lineages, thus becoming more virulent and/or easier to transmit [80].

One well-studied case of host–parasite coevolution is the interaction between the myxomatosis virus and its rabbit hosts [81]. Upon its introduction in Australia to control the rabbit population, the virus was extremely virulent, killing more than 99% of infected rabbits with a mean survival time of fewer than two weeks. After some time, less virulent virus strains emerged and spread in the rabbit population. The mortality dropped to 75–90% with an average survival time of 2.5 to 4 weeks. Most likely due to trade-offs between virulence and transmissibility, the virus had evolved to an intermediate level of virulence. For an optimal transmission, virus population densities should not be too low and host survival should not be too short, so there is time for transmission to new hosts. As the virus became less virulent, the rabbits evolved better resistance, but the resistance remained incomplete, perhaps because of the limited genetic diversity in the rabbit population.

## 5. Co-Speciation and Phylogenetic Congruence

Studies on host–parasite coevolution can help to understand the reciprocal adaptations of the parasite and the host. However, evidence for coevolution is not easy to find, in particular in long-lived organisms such as vertebrates. Usually, microbial systems, such as bacteria and their bacteriophages, are used as models for the study of coevolutionary processes [82].

Host–parasite coevolution over longer periods can result in co-speciation and congruent phylogenies of host–parasite lineages. Hence, if host–parasite phylogenies are congruent, this often suggests that some coevolutionary processes are involved [83]. However, co-speciation is neither necessary nor sufficient for pairwise coevolution and does not imply coevolution as defined as reciprocal evolutionary change [13,84]. Nonetheless, in particular for vertebrates, simultaneous phylogenetic analyses of hosts and their parasites can provide a first indication for coevolution. In addition, phenomena such as host switching and host sharing may emerge in such approaches [13,27,84,85,86].

## 6. Phylogeny and Coevolution

A phylogeny (or evolutionary tree) aims to describe how organisms are related to one another in an evolutionary context [87]. Phylogenies are based on the assumption that more closely related species or taxonomic groups are more similar to one another than to more distantly related ones and that they share a common ancestor. Phylogenetic relationships are commonly depicted as trees, but sometimes also as networks. Darwin [16] was the first to depict the evolutionary relationships of organisms in a tree, followed shortly afterwards by Haeckel [88].

Phylogenies are constructed based on differences or similarities of physical or molecular characters, or other traits, which are inherited by ancestry [89,90]. Due to technical progress in the field of molecular genetics, contemporary phylogenetic analyses or reconstructions are mostly based on nucleotide sequence data of partial or complete genomes [91].

If host species and their associated parasites are ecologically tightly linked to each other over evolutionary times, host and parasite lineages are expected to diverge more or less simultaneously and show congruent phylogenies. However, since the evolutionary trajectories of the host and the parasite are also independent and evolutionary changes might occur faster in parasites than in hosts, due to their faster life history and shorter generation times, complete congruence is rarely achieved. Cross-species transmission of parasites (switching hosts) furthermore complicates a congruent phylogenetic pattern.

**Figure 3:**
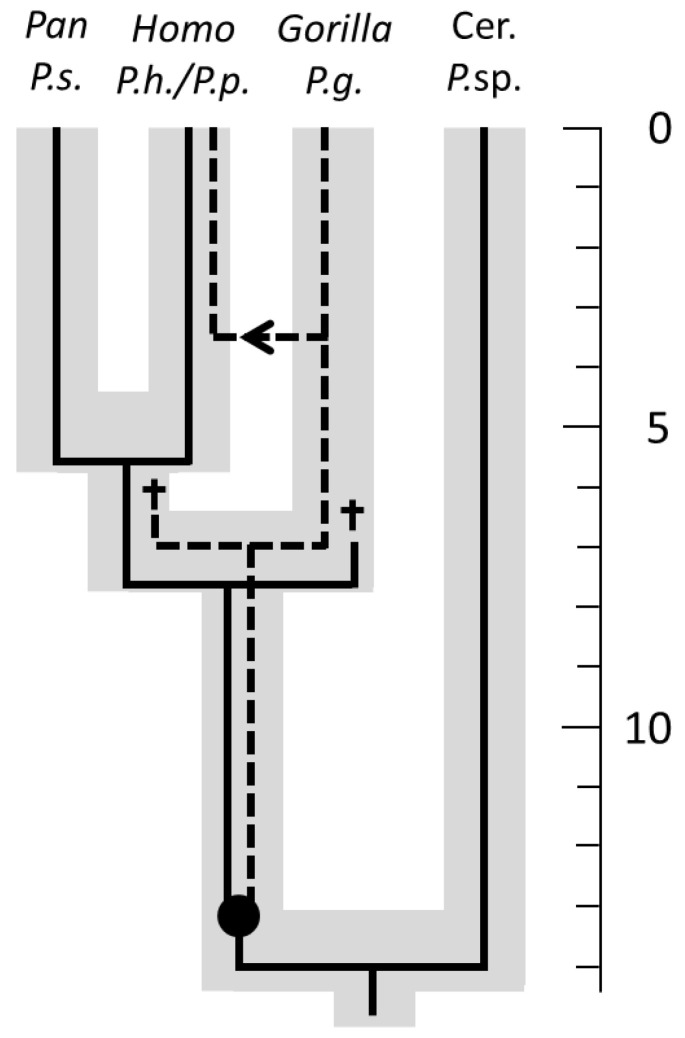
Phylogenies of Catarrhini and their lice (the host lineages are depicted in grey, the parasite lineages in black). Primate taxa: Cer. = Cercopithecidae; *Pan* = chimpanzee; *Gorilla* = gorilla; *Homo* = human; lice taxa: *P*.sp. = *Pedicinus* sp.; *P.s*. = *Pediculus schaeffi*; *P.h*. = *Pthirus pubis*; *P.g*. = *Pthirus gorillae*. Time scale on the right is in million years. The arrow indicates a host switch from gorillas to humans, the daggers indicate failed sorting (extinction or “missing the boot”), and the black circle indicates an independent parasite divergence (duplication) (modified from [92], distributed under the terms of the Creative Commons (CC) BY license).

Examples of congruent phylogenies have been reported from various host–parasite systems but also for mutualistic associations (e.g., pollination of figs by fig wasps [19,20]). An interesting example of phylogenetic congruence exists between hominids and their lice [92] (Figure 3).

In a phylogenetic study of the lice of African hominids (African apes, including humans), Reed et al. [92] found evidence for a parasite duplication ca. 13 million years ago, which led to the extant genera *Pediculus* and *Pthirus*. Humans share the genus *Pediculus* with chimpanzees and most likely also with their most recent common ancestor. Gorillas are infested by lice of the genus *Pthirus*. Interestingly, humans are also hosts to a species of the genus *Pthirus*, which they most likely received secondarily by a host switch from gorillas about 3 to 4 million years ago.

Among examples where the phylogenies of parasites and hosts match well is the case of SIV in African green monkeys [93]. Each of the five studied green monkey species is host to a monophyletic clade of SIV lineages, indicating tight coevolution and codivergence among the host and the parasite. In majority of cases, phylogenies of the host and the parasite are not as strictly correlated, but often a tendency for congruence between host and parasite phylogenies is discernible, as demonstrated by a study comparing the phylogenies of virus families with phylogenies of their eukaryotic hosts [94]. The study furthermore revealed that cross-species transmission is a near-universal feature of the investigated viruses.

## 7. Conclusions

Coevolution is an important framework to understand host–parasite interactions, and hence also infectious diseases in primates. Although the processes of co-adaptation (on a micro- and macro-evolutionary level) are often difficult to study, comparative analyses of phylogenies of parasites and their hosts can provide information on host switches and on the timeframes over which parasites and hosts interacted [95]. This can help to understand differences in virulence of the same or similar parasites in different hosts and to develop scenarios about the spread of parasites within and between populations and species.

## Data Availability

Not applicable.
